# Disease‐duration based comparison of subsets of immune cells in SARS CoV‐2 infected patients presenting with mild or severe symptoms identifies prognostic markers for severity

**DOI:** 10.1002/iid3.402

**Published:** 2021-01-16

**Authors:** Archana Kulkarni‐Munje, Sonali Palkar, Shubham Shrivastava, Sanjay Lalwani, Akhilesh C. Mishra, Vidya A. Arankalle

**Affiliations:** ^1^ Department of Communicable Diseases, Interactive Research School for Health Affairs (IRSHA) Bharati Vidyapeeth (Deemed to be University) Pune India; ^2^ Department of Paediatrics, Bharati Vidyapeeth Medical College Bharati Vidyapeeth (Deemed to be University) Pune India

**Keywords:** adaptive immune cells, disease severity, innate immune cells, PRNT50, SARS CoV‐2

## Abstract

**Introduction:**

Infection with SARS‐CoV‐2 leads to a spectrum of symptoms. Understanding the basis for severity remains crucial for better management and therapy development. So far, older age, associated‐comorbidities, and IL‐6 have been associated with severity/mortality.

**Materials and Methodology:**

As a primary step, we analyzed the frequency and functional profile of innate immune cells (NK cells/dendritic cells/monocytes) and adaptive immunity‐driving lymphocytes (B cells/T cells/follicular T helper cells) by flow cytometry. Sixty cases of SARS CoV‐2 infection (25 severe, 35 mild) and ten healthy subjects without SARS CoV‐2 IgG were included. Disease‐duration based analysis of immune profile was explored for early events differentiating the two disease forms. Neutralizing antibody titers were determined by PRNT.

**Results and Conclusion:**

Disease severity was found to be associated with impaired maturation of mDCs and hyperactivation of NK, follicular T helper cells, and CD8 T cells. Lower IL‐21 receptor expression on memory B cells indicated an imbalance in IL‐21/IL‐21 R ratio. Lower BCMA positive plasmablast cells in severe cases did suggest a probable absence of long‐term humoral immunity. Multivariate analysis revealed a progressive association of PD‐1+CD4 T cells with PRNT_50_ titers. Thus, in addition to identifying probable prognostic markers for severity, our study emphasizes the definite need for in‐depth viral antigen‐specific functional analyses in a larger patient cohort and with multiple sampling.

## Graphical abstract

Understanding pathogenesis of severity in current SARS‐CoV2 pandemic is of prime importance. Another concern is the recent observations (including ours) of early, higher neutralizing antibody titers in severe disease. India is reporting >80,000/day for several weeks now. Identification of prognostic markers for severity may help identifying “extra‐care‐needed” patients. As a first step, this flow‐cytometry based study was planned to understand (1) differences in immunologic modulations in patients with mild and severe disease (2) if we can identify early events associated with severity (3) prognostic markers for severity (4) association of functionality of immune cells with higher antibody titers in severe disease.

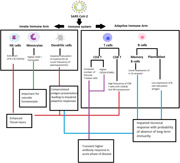

**List of abbreviations**
SARS‐CoV‐2: severe acute respiratory syndrome coronavirus 2
PRNT50: plaque reduction neutralization test at 50% reduction
IL‐6: interleukin‐6
IL‐4: interleukin‐4
IL‐10: interleukin‐10
IL‐2: interleukin‐2
NK cells: natural killer cells
IL‐21: interleukin‐21
BCMA: B cell maturation antigen (CD269); PBMC, peripheral blood mononuclear cells
COVID‐19: coronavirus disease 2019
T_FH_: follicular T helper cells
ICOS: inducible T‐cell costimulator
PD‐1: programmed death ‐1
CXCR5: C‐X‐C chemokine receptor type 5
pDC: plasmacytoid dendritic cells
mDC: myeloid dendritic Cells
TCR: T cell receptor
BAFF: B‐cell activating factor
IFN‐γ: interferon gamma
TNF‐α: tumor necrosis factor alpha
MEM: minimum essential medium
pfu: plaque forming units
ACE2: angiotensin‐converting enzyme 2
MFI: mean fluorescent Intensity
Mcl‐1: myeloid cell leukemia‐1
IQR: interquartile range
CXCR5: C‐X‐C chemokine receptor type 5 (CD185)
IL‐21R: IL‐21 receptor (CD360)

## 1 INTRODUCTION

The ongoing pandemic of SARS‐CoV‐2 has turned out to be an unprecedented threat to global public health and the economy. Irrespective of the degree of industrialization or availability of medical infrastructure, all populations have been (and are being) affected. The number of cases worldwide and corresponding mortality rates are 31.6 million and 3.7% respectively.^1^

The disease, COVID‐19, varies from an asymptomatic infection and self‐limiting mild disease to severe acute respiratory distress, multiorgan failure followed by recovery or fatal outcome. For any pathogen leading to variable clinical presentations and mortality, understanding of pathogenesis is of utmost importance. Initial studies identified older age and pre‐existing chronic conditions such as diabetes, hypertension, cardiovascular diseases, cancer, etc., as high‐risk factors for disease severity.^2^ Viral sequence variation was not related to severity^3^ and similar viral loads were detected in symptomatic and asymptomatic patients.^4^

The major focus of the scientific community has been to unravel the immunopathogenesis of the disease requiring a clear understanding of the immune response in both mild and severe disease forms. Due to the high transmissibility of the virus and biosafety concerns, studies are predominantly limited to blood investigations. An association of cytokine storm including high levels of interleukin IL‐6 production with severe disease signifies pathological immune dysregulation.^3^, ^5^, ^6^ Recent data suggest that antibody response is higher in severe disease.^7^ Though studies on innate immunity are limited,^8^ T cell and B cell responses are being actively investigated^9^, ^10^, ^11^

In India, the first COVID case was reported on 30th January 2020. As of today, (September 23, 2020) 5.73 million confirmed cases with >91,000 deaths have been reported. Older age and existing comorbidities remain the high‐risk factors. Of note, we observed significantly higher OD values in ELISA^12^ and neutralizing antibody titers in PRNT (unpublished observations) in patients with severe disease than those presenting with mild infection. In view of the need to understand immunology of the disease in general and among the Indian population in particular, we attempted to explore the functional profile of innate immune cells (monocytes, dendritic cells, and NK cells), and adaptive immune cells (B cells, follicular T helper cells, CD4 T, and CD8 T cells) in SARS CoV‐2 infected individuals presenting with asymptomatic, mild, or severe disease. Further, the dynamics of these immune cells and, relation to neutralizing antibody titers was analyzed. We found that certain earlier modulations observed during the first week of disease could differentiate between mild and severe infections.

## 2 METHODS

### 2.1 Study design and participants

This study was approved by the human ethics committee of Bharati Vidyapeeth (Deemed to be University) hospital and Medical college. A subset of patients attending Bharati Hospital, Pune, India, with confirmed positive test for SARS‐CoV‐2 reverse‐transcriptase‐polymerase chain reaction (RT‐PCR), from 20th April 2020 to 11th June 2020, was enrolled. The patients were divided into severe and mild groups according to their clinical presentation.^13^ All the patients were admitted to a special COVID facility at Bharati Hospital and research center, Pune, a tertiary care hospital.

**Figure 1:**
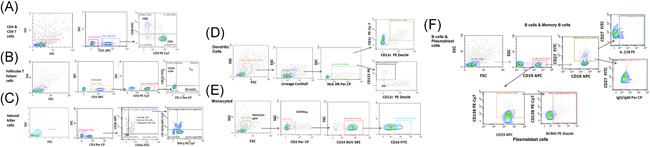
Gating strategy for the enumeration of immune cells in different study groups. Gating strategy used to distinguish different lymphocyte populations by polychromatic flow cytometry: The PBMCs from SARS CoV‐2 infected cases were stained with given number of panels of fluorochrome‐labeled antibodies to assess the frequency and functional profile of T cells (CD4 and CD8 T cells), follicular T helper cells (T_FH_), B cells, Dendritic cells, and monocytes. List of the antibodies with their clones and fluorochrome dyes is provided as Supporting Information Table. Flow cytometry data were analyzed by using Cytexpert Version 2.4. (A) CD4 and CD8 T cells‐Lymphocyte populations were identified based on forward and side scatter properties of PBMCs. The CD3 positive population was further discriminated to identify CD4 and CD8 T cells. Then CD8 T cells were analyzed for HLA DR & CD38 expression whereas the CD4 T cell population was analyzed for CD40L and IL‐2 expression. (B) T_FH_ cells‐follicular T cells were identified based on PD‐1 and CXCR5 expression on CD4 T cells as mentioned above. The T_FH_ population was further inspected for ICOS and IL‐21 expression. (c) NK cells‐Natural killer cells were identified as CD3‐CD56+CD16+/− (total NK cell population comprised cytotoxic NK cells (CD3‐CD56+CD16+) and Regulatory NK cells (CD3‐CD56+CD16‐). (D) Dendritic cells‐lymphomono gate was identified by using forward and side scatter properties of the cells. The cells were checked for Lineage cocktail (CD3, CD19, CD14, CD56, and CD20) positivity. Lineage cocktail negative cells were selected and checked for HLA DR expression. Thus, Lin‐ HLA DR+CD11c+CD1C+ cells were considered as myeloid dendritic cells (mDC) whereas Lin‐ HLA DR+CD11c‐CD123+ cells were considered as plasmacytoid dendritic cells (pDC). (E) Monocytes‐monocyte gate was recognized by FS & SS contour plot. Further, the CD3 negative cells from this population were screened for CD14 expression to identify the monocyte population. The monocyte population was further analyzed for CD16 expression to discriminate classical and nonclassical monocytes. (F) B cells and Plasmablast cells‐B cells from lymphocytes were identified with the help of CD19 expression. For further differentiation, expression of CD27 (memory B cells), IgD/IgM (class‐switched memory B cells [CSMB] and unswitched memory B cells [USMB]), and CD138 (plasmablast cells) was considered

After informed consent, approximately 5 ml of blood from all these patients and controls were collected in EDTA and processed for PBMC and plasma separation by ficoll‐histopaque based density gradient method. Plasma was used for Th1 and Th2 cytokine profiling (IFN‐γ, TNF‐α, IL‐4, IL‐10, IL‐6, and IL‐2) by cytometric bead array kit (BD Biosciences) and detection/quantitation of neutralizing antibodies (Nabs) using 50% plaque reduction neutralization test (PRNT_50_). PBMCs were subjected for immunophenotyping for selected immune cells and their subpopulations using appropriate fluorochrome‐labeled antibodies (Biolegend, San Diego & BD Biosciences) by polychromatic flow cytometry approach using CytoFLEX LX platform (Beckman Coulter). Briefly, after trypan blue‐based live cell count, PBMCs were subjected for surface staining procedure using fluorochrome‐labeled antibody cocktails (list of antibodies is provided in supplementary Information) followed by fixation (PBS with 3% paraformaldehyde). The intracellular proteins were detected after permeabilizing (BD FACs perm‐II) the fixed PBMCs with the help of appropriate fluorochrome‐labeled antibodies (Supporting Information Table). Using forward and side scatter, the lymphocyte and lympho‐monocyte populations were gated while acquiring the samples, and 50,000 gated events were recorded. The cells and the subpopulations studied are enlisted in Table 1. The gating strategy is depicted in Figure 1.

**Table 1:** Immune cell subsets analyzed in COVID‐19 cases and control subjects

No	Major cell types	Cell subsets	Subpopulation
1	T cells (CD3+ cells)	CD4 T cells (CD3+ CD4+)	IL‐2+CD4, and CD40L+CD4 T cells
		T_FH_ (CD3+CD4+CXCR5+PD‐1)	ICOS+IL‐21 +T_FH_ cells
		CD8 T cells (CD3+CD8+)	CD38+HLA DR + CD8 T cells
2	B cells	Memory B cells (CD3‐CD19+ CD27+)	Switched memory B cells (CD3‐CD19+ CD27+IgD‐)
	(CD3‐CD19+)		Unswitched memory B cells (CD3‐CD19+ CD27+IgD+)
			IL‐21 R+Memory B cells
		Plasmablast cells (CD3‐CD19+CD138+)	BCMA+plasmablast cells
3	NK cells	Total NK cells (CD3‐CD16+CD56+)+(CD3‐CD16‐CD56+)	Only CD107a +Total NK cells
			CD107a + IFN‐γ+Total NK cells
			Only IFN‐γ+Total NK cells
4	Dendritic cells	Myeloid dendritic cells (mDC) (Lineage ‐ HLADR + CD11c +CD1c+)	CD80+CD86+ mDC
		Plasmacytoid Dendritic cells (pDC) (Lineage ‐ HLADR + CD11c ‐CD123+)	CD80+CD86+ pDC
5	Monocytes	CD3‐CD14+	CD3‐CD14+CD16‐ and CD3‐CD14+CD16+

### 2.2 Determination of SARS CoV‐2 PRNT_50_

For PRNT_50_, one day before infection, 1 × 10^5^ Vero cells/ml were seeded in 24‐well plate using MEM containing 10% FBS and antibiotics. 1:5 diluted serum samples were heat‐inactivated for 30 min at 56°C. Four‐fold serial dilution was performed and mixed with an equal volume of 20‐40 pfu of SARS CoV‐2. The serum‐virus mixture was incubated for 1 h at 37°C in a humidified incubator with 5% CO_2_. After incubation, 100 µL of the mixture was added in duplicate to 24‐well plate and incubated for 1 h at 37°C in humidified incubator with 5% CO_2_. After incubation, 1 ml of 1% overlay media containing MEM, Aquacide‐II (Merck), 2% FBS and antibiotics was added to the Vero cell monolayer. Plates were incubated for 5 days at 37°C in a humidified incubator with 5% CO_2_. Five days postinfection, overlay media was discarded, cells were fixed using 3.7% formaldehyde and after washing with PBS, cells were stained using 1% crystal violet. Plates were washed and kept for air dry. Plaques were counted manually and PRNT_50_ titer was determined using Karber's formula as described earlier.^14^ Samples with PRNT_50_ titer ≥20 were considered seropositive.

### 2.3 Statistical analysis

Statistical analysis was performed using SPSS 20.0 software and Graph Pad Prism 5.0 software. Median and interquartile range (IQR) were used to describe continuous variables. Mann‐Whitney *U* test was used to compare the median for continuous variables between the study groups. For univariate and multivariate analysis, R programming was used.

## 3 RESULTS

### 3.1 Study population

The study included 60 COVID‐19 patients and 10 apparently healthy individuals negative for IgG‐anti‐SARS‐CoV2 antibodies. Patients admitted to intensive care units for oxygen/ventilator support were designated as suffering from a severe disease (SD, *n* = 25) while those with mild (*n* = 20) or no (*n* = 15, asymptomatic) symptoms were classified as MD. Among the SD patients, four succumbed to infection while 21 were discharged. Follow up blood samples collected from SD (*n* = 15) and MD (*n* = 5) patients were also studied. The demographic characteristics of the study participants are shown in Table 2. COVID‐19 cases showed a marked lymphopenia as evidenced by lower lymphocyte percentage (median‐28.83%) as compared to the control subjects (median‐61.06%) (*p* < .0001).

**Table 2:** Demographic characters of SARS CoV‐2 positive cases and control subjects

	Mild			
	Symptomatic	Asymptomatic	Severe	Control
Number	20	15	25	10
Age in years (median)	38.5 yrs	38	51	35
Sex ratio	14M/6F	4M/11F	20M/5F	5M/5F
Comorbidities (diabetes, hypertension, cardiovascular diseases, and obesity)	4/20 (20%)	3/15 (20%)	16/25 (64%)	None

To understand the contribution of major immune cell subsets in the pathogenesis of SARS CoV‐2 infection, we evaluated the frequencies of antigen‐presenting cells (dendritic cells and monocytes), natural killer cells, T cells (CD4 T cells, CD8 T cells, and follicular T helper cells [T_FH_]) and B cell subsets (memory B cells and plasmablast cells). Cytokine storm involving a major role of IL‐6 is already documented and hence we analyzed Th1 (IFN‐γ, TNF‐α, and IL‐2) and Th2 (IL‐4, IL‐6, and IL‐10) plasma cytokine levels to delineate association with these cells if any. Table 3 provides the median and IQR of different immune cell subsets with associated markers in different study groups.

**Table 3:** Comparison of Immune profiles in patients with mild or severe clinical presentation

	Immune parameters	Mild (*N* = 35)	Severe (*N* = 25)	Mild versus severe (Mann–Whitney *U* test)	Controls (N = 10)	Mild versus Controls (Mann–Whitney *U* Test)
	Median (IQR)	Median (IQR)	p value	Median (IQR)	*p* value	
Innate immune response	Myeloid DC % Total	0.43 (0.19–0.86)	0.21 (0.02–0.65)	0.056	0.61 (0.37–0.73)	0.56
	mDC (CD80+ & CD86 +) %	87.42 (84.2–90.68)	56.89 (41.57–77.59)	<0.0001	88.52 (86.4–92.13)	0.28
	CD80 MFI mDC (CD80+ & CD86)	8976(7747–10795)	8364 (6591–11,277)	0.333	9403 (8235–10583)	0.67
	CD86 MFI (CD80+ and CD86+)	6265(5127–8107)	2708 (2144–4829)	0.001	8628 (7695–9996)	0.01
	Plasmacytoid DC % Total	0.17(0.10–0.26)	0.07 (0.02–0.21)	0.005	0.37 (0.28–0.54)	0.01
	CD80+ pDC %	57.27(40.08–67.1)	42.55 (19.05–73.68)	0.164	67.96 (51.94–85.98)	0.05
	CD80 MFI (pDC)	6025(5293–8874)	7897 (6318–12284)	0.047	6555 (6222–7968)	0.17
	CD86+ pDC %	24.75(13.77–37.49)	20.83 (0–43.81)	0.692	18.4 (9.59–41.11)	0.86
	CD 86MFI (pDC)	390.6(352–578.3)	622 (445.7–775.1)	0.863	524 (319–564.6)	0.6
	Monocyte % Total	1.925(0.57–2.98)	1.74 (0.65–3.56)	0.872	1.71 (0.8–3.56)	0.97
	CD16+ monocyte %	11.96(6.91–16.66)	17.87 (5.53–25.73)	0.301	4.91 (4.45–8.44)	0.02
	Total NK%	8.52(4.76–12.4)	7.74 (5.105–18.24)	0.567	7.08 (3.42–10.68)	0.76
	IFN‐γ & CD107a+ total NK%	0.06(0–0.32)	1.17 (0.09–4.88)	0.001	1.11 (0–3.43)	0.05
	Only CD107a+ total NK%	11.69(6.79–24.39)	18.03 (9.71–31.25)	0.305	19.71 (8.46–27.7)	0.34
	Only IFN‐γ + total NK%	0(0‐0.038)	0 (0–2.4)	0.007	0 (0–0.63)	0.2
T cell response	CD8%	32.04(22.3–41.45)	30.64 (25.63–40.54)	0.792	34.62 (26.63–40.29)	0.42
	HLA DR and CD38 + CD8%	0.13(0.02–0.78)	2.12 (0.61–8.36)	0.0001	0.08 (0.01–0.11)	0.2
	CD4%	60.93(49.53–68.25)	54.9 (41.32–66.2)	0.215	53.44 (47.89–61.45)	0.18
	CD40L+ CD4%	6.09(3.305–11.97)	8.04 (6–13.82)	0.356	4.58 (3.35–7.93)	0.43
	CD40L MFI	21399(20164–23105)	24397 (22559–25459)	<0.0001	19,521 (19,134–20,993)	0.01
	IL‐2 + CD4%	0.02(0.01–0.06)	0.205 (0.08–1.68)	<0.0001	0.01 (0–0.07)	0.01
	IL‐2 MFI	2425(1983–4385)	1769 (1637–2076)	0.071	2247 (2171–4494)	0.61
	CD4/CD8	1.8(1.165–3)	1.7 (1.1–2.54)	0.587	1.43 (1.12–1.77)	0.21
Follicular T helper cell Response	T_FH_ %	0.09(0.02–0.22)	1 (0.525–1.845)	<0.0001	0.1 (0.03–0.24)	0.79
	CXCR5+CD4%	14.62(9.59–20.03)	23.43 (12.46–41.77)	0.031	13.58 (10.38–16.52)	0.96
	PD‐1+ CD4%	0.34(0.155–1.39)	1.84 (0.67–3.01)	0.009	0.29(0.2‐0.54)	0.88
	ICOS and IL‐21 + cells %	75(50–100)	71.09 (59.33–79.87)	0.546	56(42.86‐83.67)	0.71
	Il‐21 MFI	14979(13186–21545)	18814 (15943–22635)	0.015	15223(13559–19936)	0.28
	ICOS MFI	9384(7512–13657)	10708 (9177–14010)	0.12	9603 (7954–11591)	0.42
B cells	B cells %	10.89 (8.53–16.47)	6.02 (2.15–17.1)	0.134	13.6 (5.2–16.3)	0.22
	Memory B cells %	23.22 (14.42–31.8)	36.04 (21.73–43.01)	0.01	26.9 (15.23–39.68)	0.52
	Class switched memory B cells %	90.65 (81.08–93.67)	90 (84.14–94.52)	0.741	89.35 (80.02–96.37)	0.83
	Unswitched memory B cells %	7.82 (5.7–17.08)	10 (4.79–15.2)	0.952	12.63 (5.23–16.78)	0.7
	IL‐21 R + memory B cell %	6.36(1–35.69)	7.85 (2.36–29.65)	0.533	8.65 (3.68–23.56)	0.21
	IL‐21 R MFI	865.4(325.2–2541)	1324 (457.2–3548)	0.645	2123 (1632–2152)	<0.0001
Plasmablast cells	Plasmablast cells %	9.9(3.8–12.25)	10.72 (3.78–14.57)	0.852	3.21 (1.01–6.23)	<0.0001
	BCMA + plasmablast cells %	26.09(15.85–30.43)	14.7 (8.95–27.53)	0.072	25.87 (21.06‐28.3)	0.76
	BCMA MFI	29989(14346–34433)	30,816 (18,297–41,842)	0.724	28,965 (26,771–43,781)	0.67
Plasma Th1 cytokine level (pg/ml)	IFN‐γ	0.32(0–1.54)	0.43 (0.12–0.83)	0.953	0.29 (0–1.17)	0.15
	TNF‐α	0(0–0.24)	0 (0–0.24)	0.733	0.24 (0–0.85)	0.8
	IL‐2	0.155(0–0.5)	0.22 (0–0.39)	0.952	0 (0–0.25)	0.06
Plasma Th2 cytokine level (pg/ml)	IL‐10	0.465 (0.08–1.37)	2.53 (0.67–16.9)	0.0004	0.39 (0.24–0.65)	0.12
	IL‐6	4.22 (2.05–6.80)	33.78 (4.36–257.5)	0.001	1.03 (0–2.65)	<0.0001
	IL‐4	0.68 (0.31–1.32)	0.43 (0–0.89)	0.121	0 (0–1)	0.01

### 3.2 Mild symptomatic versus asymptomatic COVID‐19

Initially, we compared mild symptomatic and asymptomatic individuals with respect to all the parameters examined. Except for TNF‐α levels, the other parameters were comparable among these patient groups. TNF‐α was detected in the asymptomatic patients (median‐0.125 pg/ml [0–0.45]) while this cytokine was undetectable in the symptomatic cases (*p* = .027). Therefore, the two groups were combined (MD) while comparing with the patients with severe disease (SD).

### 3.3 Compromised antigen presentation in COVID‐19 and hyperactivation of NK cells

**Figure 2:**
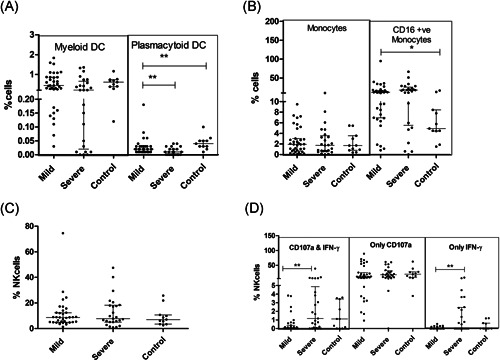
Innate immune response in relation to COVID‐19 disease severity: Vertical scatter plots denote the comparisons of frequencies of innate immune cells and their subpopulations among different study groups: (A) Frequencies of dendritic cell subsets. (B) Monocyte frequencies. (C) NK cell population. (D) Activated NK cell (Mann–Whitney *U* test; Error bars‐median and IQR). IQR, interquartile range

DC population was significantly affected by the virus infection. The proportion of total mDCs was not different among the two groups at *p* = .056 level. SD patients exhibited a lower frequency of mDCs with the mature phenotype (CD80 and CD86 mDCs) than recorded in the MD group (*p* < .0001). CD86 expression density was significantly lower in MD patients reducing further in the SD category (*p* < .001 for both). Plasmacytoid DCs were lower in both MD and SD patients, severe category exhibiting further reduction (*p* < .005). In the SD patients, pDCs showed lower expression of the maturation marker, CD80 (*p* = .047). Thus, the disease induced a strident defect in the maturation of the dendritic cell population that was aggravated in severe disease (Table 3; Figure 2A).

Although monocyte frequencies were intact, a large subset of monocytes was found to be CD16+, that is, nonclassical monocytes (Table 3; Figure 2B). While the total NK cell pool was unaffected, degranulation phenotype along with IFN‐γ was augmented in the SD patients as compared to MD cases (*p* = .001; Table 3; Figure 2C,D)

### 3.4 Hyperactivation of T_FH_ and CD8 T cells

**Figure 3:**
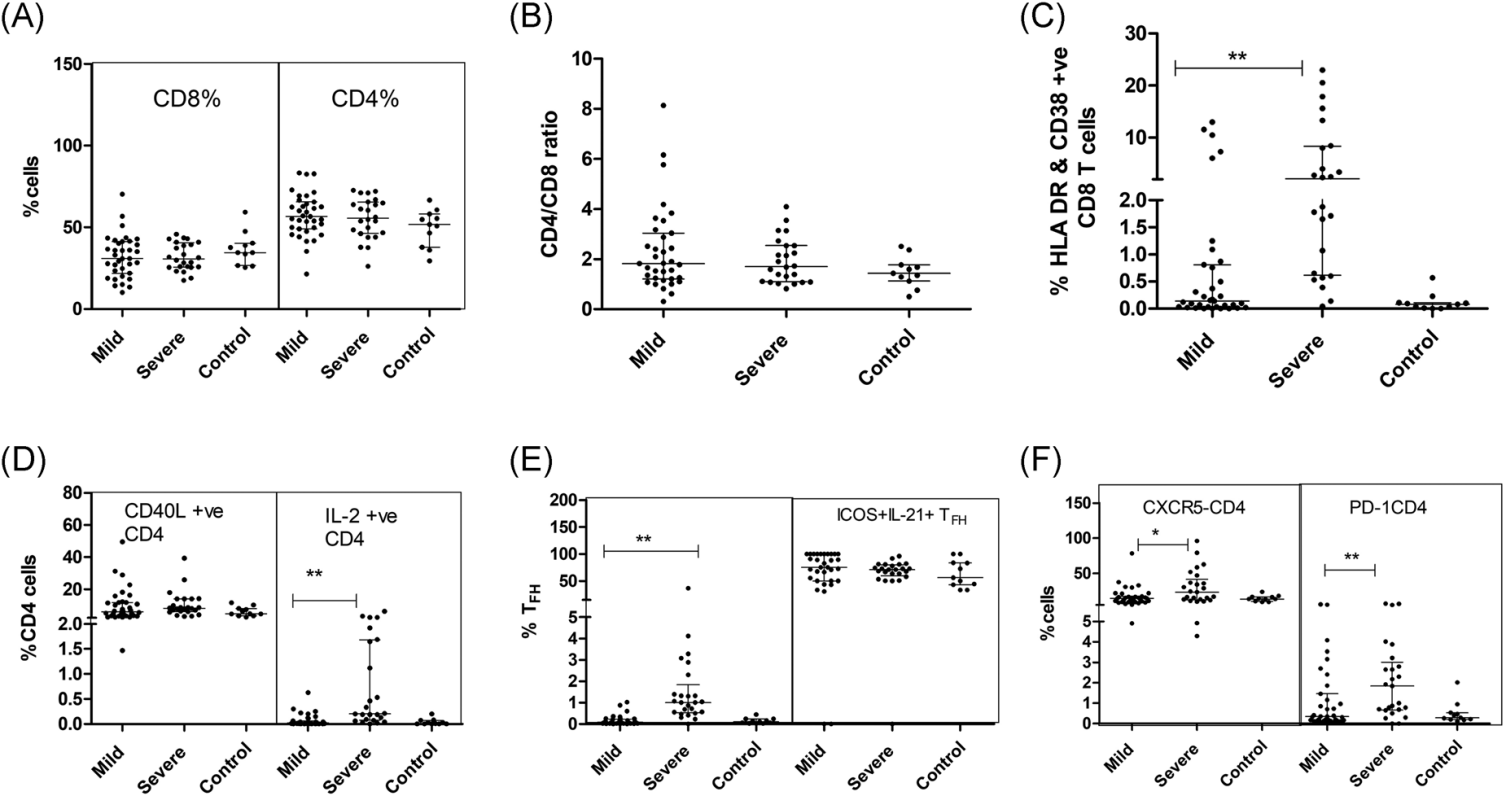
Adaptive immune response in relation to COVID‐19 disease severity (T cells): Vertical scatter plots denote the comparisons of frequencies of adaptive immune cells and their subpopulation among different study groups: (A) CD4 and CD8 frequencies (B) CD4/CD8 ratio. (C) CD8 T cell compartment with HLA DR and CD38 expression. (D) Frequency of CD40L positive CD4 T cells. (E) Follicular T cells (T_FH_) cells and T_FH_ cells with ICOS and IL‐21 expression. (F) CXCR5+CD4+ T cells and PD‐1+CD4+T cells. (Mann–Whitney *U* test; Error bars, median and IQR). IQR, interquartile range

In contrast to the recent findings of significant depletion of T cells in SARS Cov‐2 infection,^15^ T cell frequencies and CD4/CD8 ratio remained unchanged among both the patient groups (Table 3; Figure 3A,B). Although the CD8 T cell compartment was unchanged with respect to quantity, activation of these cells was evident by higher expression of HLA DR and CD38 expression in the SD patients than the MD group(*p* = .0001) (Table 3; Figure 3C).

In MD patients, CD4 T cells exhibited higher expression of CD40L (*p* = .009) that was further enhanced in the SD category (*p* < .0001). The proportion of IL‐2+ CD4 T cells was higher in the SD patients while the levels among MD patients and controls were comparable (Figure 3D). In SD patients, the proportion of T_FH_ (CXCR5 + PD‐1+ CD4+ T cells (*p* = .0001), CXCR5+CD4+PD‐1‐ T cells (*p* = .03), and PD‐1+CD4 T cells (*p* = .009) was higher than in the MD group. However, IL‐21 and ICOS expression by these subsets remained comparable between SD & MD patients (Table 3; Figure 3E,F).

### 3.5 Quantitative and qualitative loss of B cell compartment

**Figure 4:**
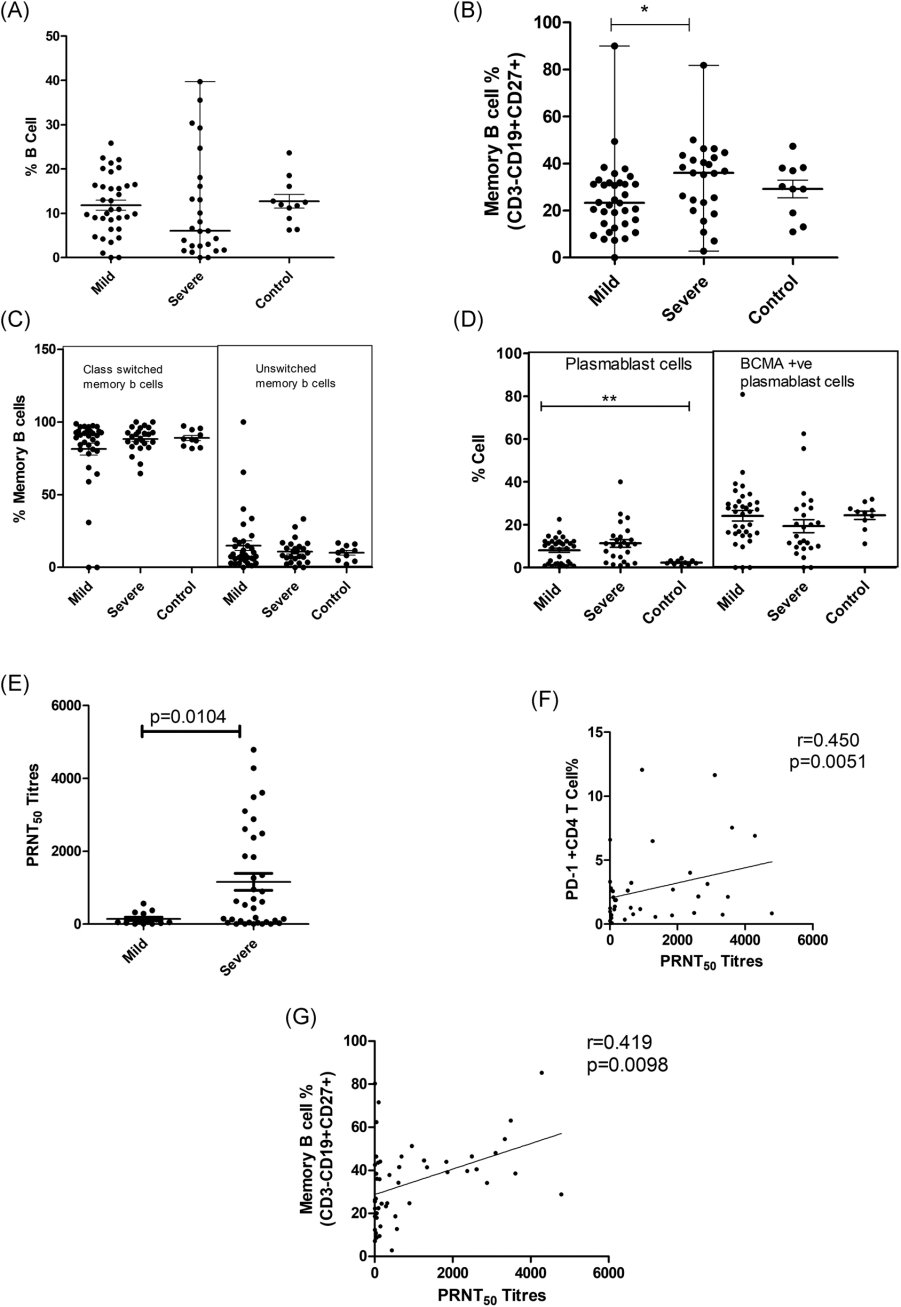
Adaptive immune response in relation to COVID‐19 disease severity (B cells and plasmablasts): Vertical scatter plots denote the comparisons of frequencies of B cells and plasmablast populations and their subpopulations among different study groups: (A) B cells, (B) Memory B cells, (C) Class switched and unswitched memory B cells, (D) Plasmablasts cells & BCMA+plasmablast cells, (E) SARS CoV‐2 PRNT50 titers in mild (median: 53.05; IQR: 22.49–294.8) and severe cases (median: 571.1; IQR: 50.31–2247) within 15 days of disease onset. (F) Association of SARS CoV‐2 PRNT_50_ titers (15 days of disease onset) with PD‐1+CD4 T cells. (G) Association of SARS CoV‐2 PRNT_50_ titers (15 days of disease onset) with memory B cells. (Mann–Whitney *U* test; Error bars, median and IQR). IQR, interquartile range

Although the total B cell frequency remained unchanged, the SD patients exhibited a higher frequency of memory B cells than the MD group (Table 3; Figure 4A,B). The class switched and unswitched memory B cells percentages were comparable among all the groups (Table 3; Figure 4C). Even though the frequency of IL‐21R positive memory B cells was unchanged, the IL‐21 receptor expression intensity (MFI) was lower in MD patients when compared to the controls (*p* < .0001). No difference was recorded among MD and SD groups. In addition, a sharp rise in plasmablast cells was evident in the MD patients (*p* < .0001) that remained comparable in the SD group. (Table 3; Figure 4D)

### 3.6 Plasma cytokine profile: An indication of immune paralysis

**Figure 5:**
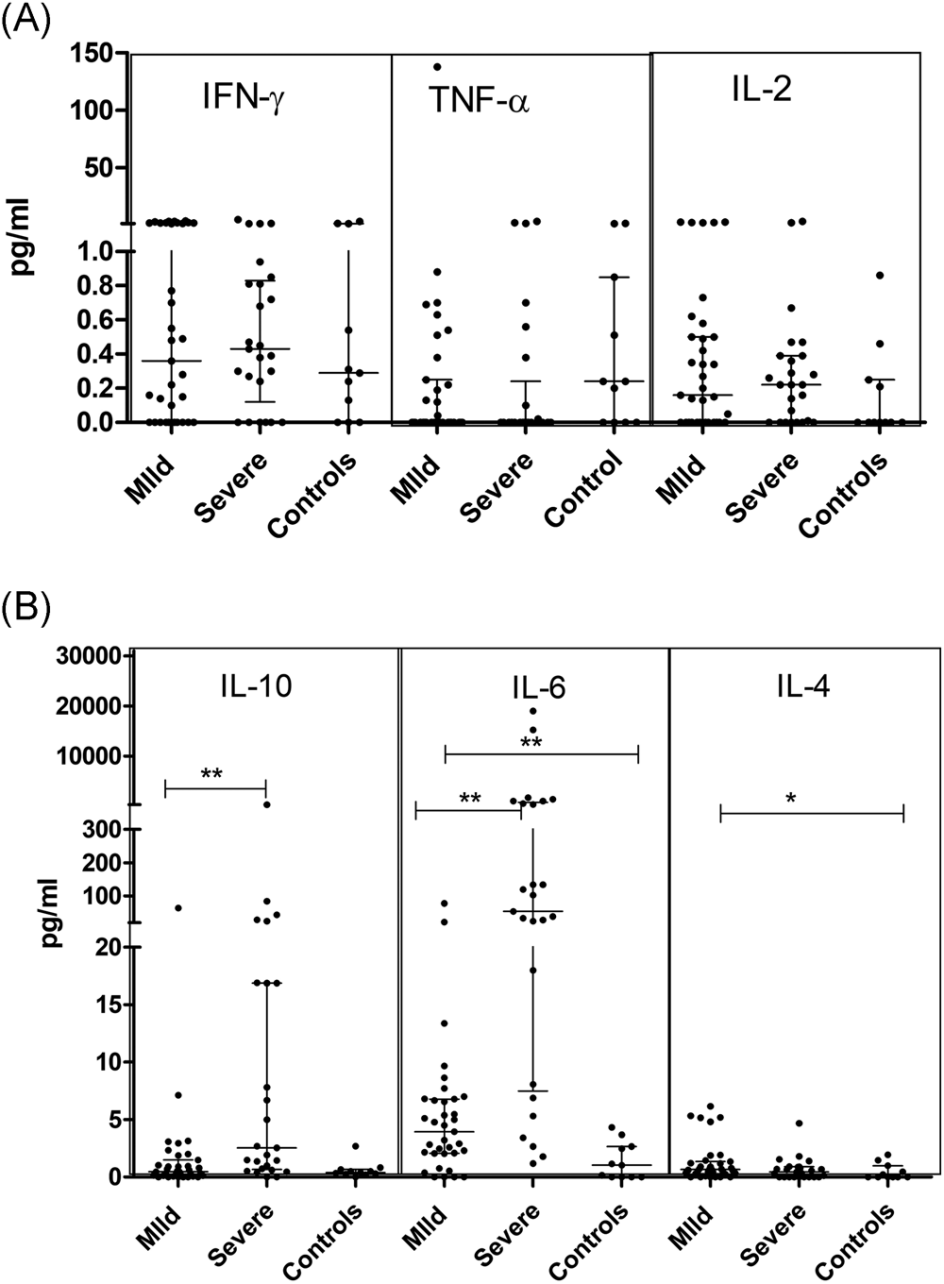
Plasma Th1/Th2 Cytokine levels in SARS Cov‐2 Infection: (A) Th1 cytokine profile (IFN‐γ, TNF‐α, and IL‐2). (B) Th2 cytokine profile (IL‐10, IL‐6, and IL‐4; Mann–Whitney *U* test; Error bars, median and IQR). IQR, interquartile range

In line with previous findings, plasma cytokine profiling revealed the dominance of IL‐6 secretion in COVID‐19 patients (*p* = .001) which was more pronounced in severe cases than in mild disease (*p* = .001). IL‐10 levels were also higher in severe disease (*p* = .0004). Only mild disease showed a significant rise in IL‐4 (*p* = .01) as compared to the controls (Table 3; Figure 5A,B).

In view of the observed decline in the frequency of myeloid dendritic cells, we compared cytokine profiles of MD and SD patients with or without mDC reduction. Analyses revealed that in the mild patients, plasma IL‐4 levels increased with rise in mDC frequency (*p* = .023). No such difference was recorded in the SD patients.

#### 3.6.1 Neutralizing antibody titers in relation to the parameters investigated

In accordance with our earlier observations, severe disease was characterized by higher neutralizing antibody titers. During the first 2 weeks, PRNT_50_ titers were significantly higher in the SD patients (median: 571.1) than the MD group (median: 53.05, *p*= .010; Figure 4E). Therefore, the proportions and effector functions of immune cells and cytokines were compared in relation to neutralizing antibody titers in these groups. In univariate analysis, CD86+ pDC (*p* = .017), PD‐1CD4 (*p*=.0051; Figure 4F) and memory B cells (*p* = .00982; Figure 4G) correlated with PRNT_50_ titer. However, in multivariate analysis, PD‐1+CD4 emerged as the single variable influencing PRNT_50_ titers (*p* = .003, *R* ^2^ = 0.421). As mentioned earlier, PD‐1 expression on CD4 T cells was higher in severe disease.

#### 3.6.2 Relationship of disease duration and modulation of parameters examined in the SD and MD patients

Next, we compared the proportion of immune cells and cytokine concentrations in the MD and SD patients at different time points after the onset of clinical symptoms (Table 4). These comparisons revealed the following patterns in the SD patients: (1) Lowering of activated mDCs (CD80+ and CD86+) and increase in T_FH_ cells that continued till the 3rd‐week postonset; (2) lower pDCs and a marginal reduction in B cells during the 2nd week (*p* = .061) and higher IL‐2+CD4 cells during the first two weeks; (3) difference only in the first week; increase in HLA DR & CD38+ CD8 and memory B cells and decrease in BCMA + plasmablast cells; (4) modulation during 2nd week, decrease in CD16+ Monocytes and reduction in total NK cells.

**Table 4:** Analysis of immune cell frequencies/cytokines in relation to disease severity and duration

Name of Immune cell subset	Type of illness	Week 1	Mann–Whitney *U*	Week 2	Mann–Whitney *U*	Week 3	Mann–Whitney *U*	Week 4	Mann–Whitney *U*
mDC (CD80+ and CD86+) %	Mild	87.6	0.007	87.8	0.001	87.7	0.013	87.8	1
	Severe	31.77		33.33		56.02		83.35	
Plasmacytoid DC %	Mild	0.17	0.012	0.11	0.032	0.15	0.276	0.13	0.121
	Severe	0.06		0.03		0.01		0.06	
CD4%	Mild	56.8	0.19	59.3	0.753	57.08	0.355	58.12	0.121
	Severe	71.08		63.7		60.64		10.735	
T_FH %_	Mild	0.15	0.001	0.15	0.012	0.17	0.045	0.16	0.121
	Severe	1.29		1.07		1.04		5.77	
Il‐21 MFI (T_FH_)	Mild	18836	0.28	14852	0.038	16471	0.165	15160	0.121
	Severe	21875		21751		17500		15178	
CD16+ monocytes %	Mild	30.37	0.165	10.16	0.025	10.16	1	10.16	1
	Severe	13.22		17.86		12.39		18.3	
Total NK %	Mild	7.6	0.316	6.2	0.042	6.38	0.355	6.29	1
	Severe	2.88		2.65		3.03		8.866	
HLA DR + CD38 + CD8%	Mild	0.495	0.041	0.16	0.414	0.29	0.122	0.18	1
	Severe	3.69		2		3.03		0.405	
IL‐2 + CD4%	Mild	0.13	0.005	0.03	0.016	0.06	0.06	0.035	1
	Severe	0.49		0.47		0.3		0.015	
B Cells %	Mild	13.44	0.033	10	0.061	13.01	0.64	11.5	0.439
	Severe	4.63		6.93		7.47		13.66	
Memory B cells %	Mild	14.3	0.001	25.76	0.116	31.26	0.355	28.73	0.121
	Severe	41		42.86		34.78		42.84	
Class Switched memory B cells %	Mild	81.15	0.589	89.31	0.586	88.22	0.537	88.76	0.439
	Severe	85.23		86.53		90.02		81.73	
Unswitched memory B cell%	Mild	18.27	0.643	10.27	0.66	10.97	0.436	10.28	0.425
	Severe	14.03		12.61		10		16.58	
BCMA+plasmablast cell %	Mild	23.7	0.02	21.32	0.136	23.56	0.563	25.32	0.935
	Severe	13.23		10.25		12.34		21.25	
BCMA MFI	Mild	25326	0.82	34215	0.996	27896	0.036	36547	0.865
	Severe	21457		26359		21789		39875	
IL‐6 pg/ml	Mild	6.14	0.939	5.12	0.368	9.68	0.105	5.83	1
	Severe	5.82		27.36		49.13		21.1	

Note‐The week wise disease duration is calculated after the onset of symptoms.

### 3.7 Immune cells/cytokines at the time of first and last sampling in the COVD19 patients

**Figure 6:**
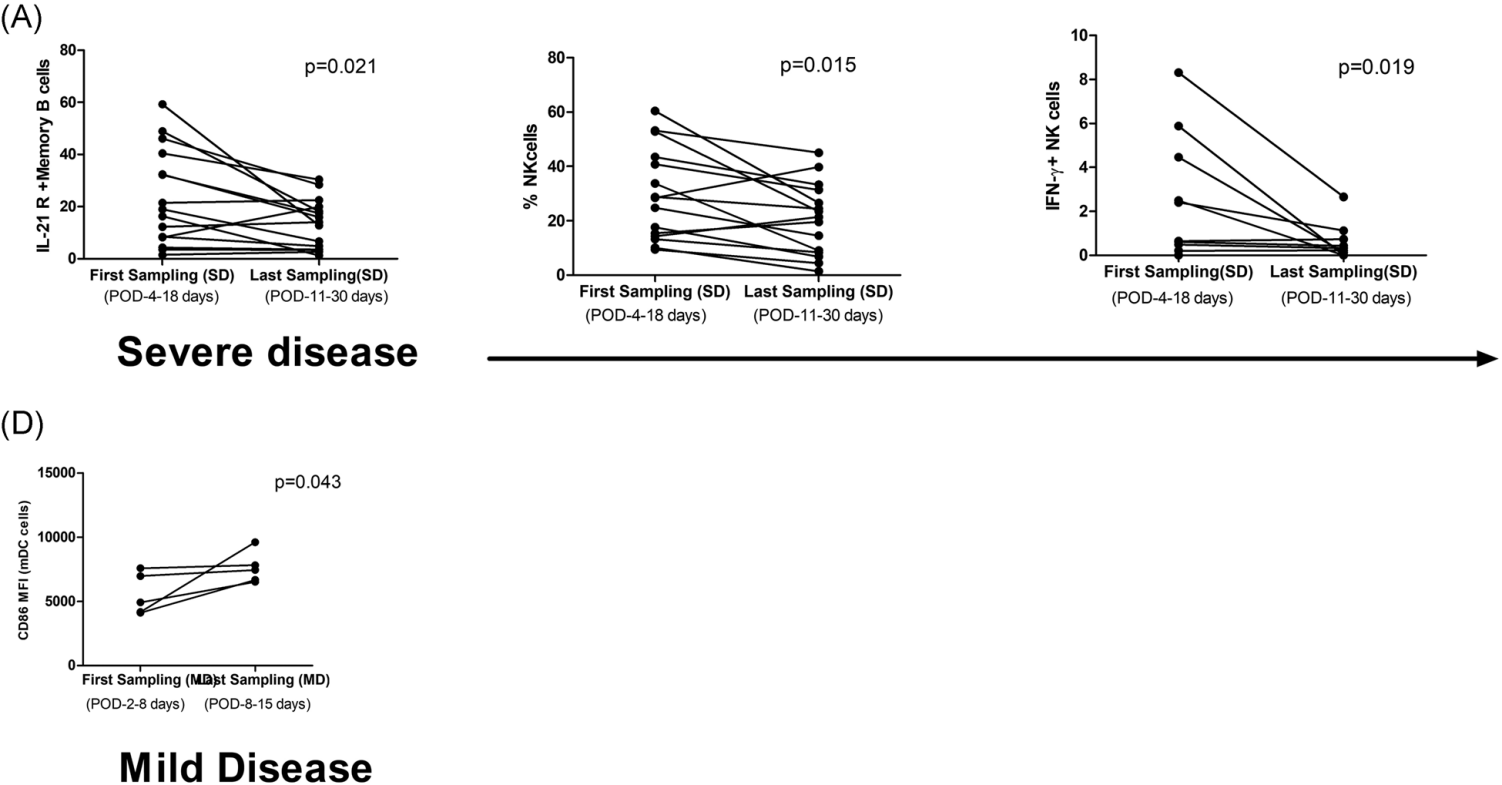
Immune profiles in SD (A–C) and MD (D) patients at first and last sampling: Reduction in (A) Il‐21R positive memory B cells, (B] NK cell frequency, and (C) IFN‐γ positive NK cells. In the MD group (D) CD86 expression by myeloid DCs increased (Wilcoxon signed‐rank test).

Follow up samples were available from 15 SD and 5 MD patients. A significant decline in IL‐21R positive memory B cells (*p* = .021) and NK cell function in terms of degranulation (*p* = .015) and IFN‐γ secretion (*p* = .019) capacity of NK cells was noted in the SD patients (Figure 6A,B,C). In the MD patients, except for the rise in CD86 MFI on myeloid DCs (*p* = .043), other immune cells and cytokines remained unchanged (Figure 6D).

## 4 DISCUSSION

This study reports alterations in the circulating immune cells and cytokines associated with innate and adaptive immune responses in COVID‐19. We first compared all the MD and SD patients irrespective of the duration of disease onset (Table 3). However, when the same parameters were compared with respect to disease duration and severity, differential modulations were obvious and are discussed here.

### 4.1 Innate immune response

So far, understanding of innate immune response to SARS CoV‐2 is limited.^8^ We assessed the proportions and effector functions of three important cell populations of the innate immune system, that is, dendritic cells, monocytes, and NK cells. Interestingly, a reduction in dendritic cells was found in the SD patients (Table 4). Comparison in relation to disease duration revealed an association of early (first week) reduction of mDCs with the mature phenotype (CD80 and CD86) and total pDCs with severe disease that continued for at least 2 weeks (Table 4). Interestingly, patients with lower frequency of mDCs exhibited lower plasma IL‐4 levels. Role of IL‐4 in the process of maturation of dendritic cells is well documented.^16^ For the first time, we show that early modulation of dendritic cells is associated with disease severity and could play a crucial role in COVID‐19 pathogenesis. In‐depth evaluation in terms of utility as a prognostic disease severity marker and treatment is warranted.

Involvement of monocytes, a key cell of the innate system, is documented in COVID‐19.^17^ Monocytes from peripheral blood of healthy donors were shown to be positive for ACE2 receptor and could be infected by SARS‐CoV‐2.^18^ In contrast to previous reports,^17^ the proportion of monocytes were not different in MD and SD patients. Nonetheless, nonclassical monocytes (CD16+) did increase in both the patient categories. During the second week of the disease, SD patients exhibited significantly higher number of nonclassical monocytes than the MD group that could have led to further enhancement of proinflammatory cytokine secretion and worsening of the disease.

NK cells are one of the first cells to protect the host from viral infections. Lowering of these cells has been reported in COVID patients.^19^ In our patient series, NK cell frequencies were generally lower in SD patients reaching a significant difference during the second week. Increased frequency of activated NK cells (CD107a and IFN‐γ expression) in SD patients is noteworthy and may be responsible for the aggravation of the infection by providing an inflammatory milieu.

### 4.2 T cell responses in SARS CoV‐2 infection

T cell activation remains an essential event in effective cellular immune response against a variety of viral infections. In COVID‐19, reduction,^15^, ^20^, ^21^ as well as unchanged^21^ frequencies of CD4 and CD8 T cells, have been reported. Our study did not find any alteration in CD4 and CD8 T cell frequencies in both patient categories resulting in unaltered CD4/CD8 ratios. However, a substantial increase in the activated CD8 T cells (HLA‐DR and CD38+) in SD patients alone indicates the role of hyperactivation of CD8 T cells in disease severity. Of note, the difference was striking during the initial phase (first week) suggestive of a crucial role in disease pathogenesis. Prospective studies are needed to ascertain the utility of HLA‐DR/CD38 positive CD8 T cells as a prognostic marker for disease severity. Following immunization with attenuated yellow fever and smallpox vaccines as models for acute viral infection, Miller et al.^22^ demonstrated that CD8 T cells with overexpression of HLA‐DR and CD38/Ki67 markers reflect virus‐specific CD8 T cells.

While confirming higher frequency of IL‐2 + CD4 T cells in severe disease,^23^ our study revealed higher expression of IL‐2 by CD4 T cells in SD patients during the first week of illness (Table 4) suggestive of an important role in pathogenesis and possibility of being a prognostic marker of disease severity. Association of elevated IL‐2 levels with disease severity is known for other coronaviruses such as SARS.^24^, ^25^, ^26^

T cells of SD patients exhibited higher percentage of PD‐1+ CD4 T cells that are characterized by poor effector function, sustained expression of inhibitory receptors, and reduced TCR sensitivity.^27^ IL‐10, an inhibitory cytokine, not only prevents CD4 T cell proliferation but also can induce T cell exhaustion.^28^ Indeed, the SD patients showed higher plasma IL‐10 levels which are speculated to be responsible for functional exhaustion of T cells.

Though we did not study virus‐specific response, expression of CD40L on CD4+ T cells does indicate sensitization of these cells in the SD patients that could be linked to the secretion of proinflammatory cytokines. Notably, hydroxychloroquine, one of the treatments for COVID, can attenuate progression to severe disease by repressing CD40L expression on activated T cells.^29^ Taken together, CD40L seems to be playing a critical role in COVID‐19 pathogenesis. We observed higher CD40L expression by CD4 T cells in the SD patients. CD40L plays an important role in the germinal center formation.^30^ In this context, higher PRNT50 titers in the SD group are noteworthy. However, when MD and SD patients were compared with respect to disease duration, no significant difference emerged suggesting the involvement of additional factors.

### 4.3 Cellular factors responsible for humoral response

Though long‐term follow up data is not yet available, a rapid decline of IgG‐anti‐SARS‐CoV‐2 antibodies along with the possibility of reinfection is suggested.^31^ On the other hand, severe disease has been associated with higher antibody titers (including neutralizing).^7^ Taken together, the dual role of antibodies in protection/pathogenesis of SARS‐CoV‐2 is not clear and needs immediate attention. As our own data identified the association of early and higher neutralizing antibodies with disease severity, we determined characteristics of key cellular players of humoral responses, that is, B cell and follicular T helper cell compartments in mild and severe disease.

While confirming a significant decline in B cell population in the SD patients^3^ and patients with longer viral RNA shedding,^11^ our data revealed that this difference was evident in the first week of disease. B cell sequestration in secondary lymph nodes leading to an early enhanced humoral immune response in severe cases seems a distinct possibility.

The germinal center formation is one of the major steps in the generation of virus‐specific antibodies. Generally, T_FH_ cells reside at lymph nodes or secondary lymphoid organs, but, in severe SARS CoV‐2 infection, we observed an elevated frequency of T_FH_ in peripheral blood suggestive of their hyperactivation. Moreover, IL‐21 secretion was also higher in severe cases. IL‐21 exerts an array of its actions through IL‐21R expressed on B cells for the induction of affinity maturation and somatic hypermutation.^32^ Though the frequency of total memory B cell pool in SD cases was higher, these cells were equipped with lower IL‐21 R expression. Thus, although IL‐21 is expressed in abundant amount, due to the lack of receptor availability, hampering processes like affinity maturation and differentiation into antibody‐secreting cells seems likely. Moreover, unused IL‐21 may contribute to higher soluble plasma IL‐21 levels and, being a member of the Th‐17 family may aggravate lung injury in severe cases through cytokine storm mediated immune paralysis.

Multiple regression analysis signified an association of PD‐1+CD4 T cells with neutralizing antibody titers that correlated with disease severity. Although PD‐1 is an exhaustion marker expressed on activated CD4 and CD8 T cells, it reduces TCR ligand sensitivity imposing a more stringent selection threshold for competing B cells.^33^ It also promotes affinity maturation, improving the quality/neutralization capacity of antibodies in secondary lymph nodes. Thus, PD‐1 expression by CD4 T cells seems to have a dual influence in COVID‐19 pathogenesis.

Memory B cells formed during primary infection can induce long‐lasting plasma cells to protect the host from recurrent infection. B cell maturation antigen (BCMA) expression is linked to the long lifespan of plasma cells.^34^ Activation of BCMA by BAFF (B cell–activating factor) increases expression of the antiapoptotic molecule myeloid cell leukemia‐1 (Mcl‐1), pointing to the mechanistic basis by which the BAFF/BCMA axis promotes long‐term survival of plasma cells.^35^ We observed an early, higher frequency of memory B cells in the SD patients along with a lower proportion of BCMA + plasmablast cells. Memory B cells should be able to generate a protective immune response after reinfection while a low number of BCMA + plasma cells in SD patients may lead to rapid decline over time. Dynamics of antibodies over at least one year in relation to disease severity along with a simultaneous evaluation of relevant immunologic mechanisms will be able to address the most relevant public health issue of protection from SARS‐CoV‐2 exposure/re‐exposure.

After recognition of IL‐6 as the prognostic marker for severity in COVID‐19,^5^, ^6^, ^36^ several groups including ours (this study) confirmed this observation. In fact, anti‐IL‐6 antibodies are being used in the treatment of SD patients. In accordance with acute inflammatory response, both IL‐6 and IL‐10 levels increased in SD patients while IL‐4, an anti‐inflammatory cytokine predominated in mild disease helping prevention of progression to severe disease. Relation of IL‐4 levels with frequency of mDCs is noteworthy. Importantly, IL‐4 is observed to reduce the expression of the ACE2 receptor and may partially contribute to restraining viral entry.^37^

Our study has a few limitations. The sample size was limited and very few samples were available in the 4th week of disease. Due to lymphopenia and restricted sample volume, the study was limited to a few markers. Nonetheless, the results form the basis for planning in‐depth analyses involving larger cohort and sequential follow up. Our results warrant exploring the role of IL‐21/IL‐21R expression ratio (memory B cells), CD40‐CD40L interaction (CD4 T cells & B cells), and kinetics of BCMA expression on plasma cells in addressing crucial involvement of humoral response in SARS CoV‐2 pathogenesis and protection.

**Figure 7:**
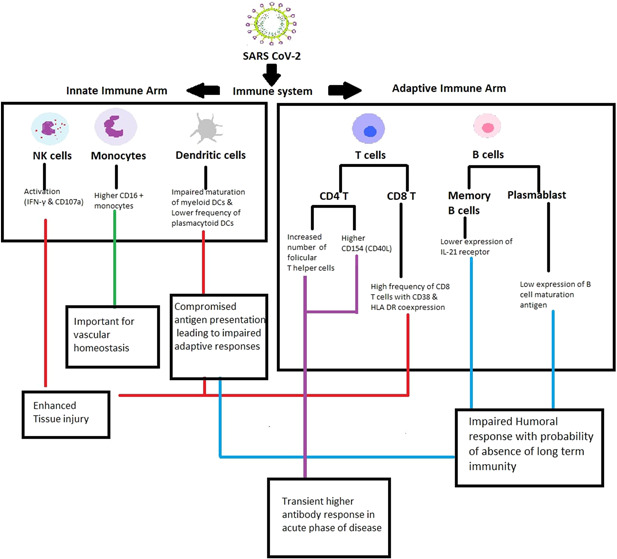
Schematic presentation of the major findings: Multiparametric flow cytometry was used to enumerate immune cells from innate ((NK cells, DCs, and monocytes and adaptive (T cells and B cells) immunity arms in COVID‐19 patients presenting with mild or severe disease. The findings pointed out major defects in the maturation (CD80 and CD86 expression) of myeloid dendritic cells and reduction in plasmacytoid dendritic cell frequency, emphasizing compromised antigen presentation. Enhanced frequency of nonclassical monocytes could be an important event in maintaining vascular homeostasis in the inflammatory cytokine milieu formed due to the infection. Activated NK and CD8 T cell subsets observed in severe cases may enhance lung tissue injury and disease severity. Likewise, activation of T_FH_, and CD4 T cell compartments in these cases may lead to a transient higher humoral response observed during the acute phase of infection. However, whether these antibodies are protective or aid in disease severity remains unclear. Lower IL‐21 receptor expression on B cells may lead to impaired proliferation and differentiation of B cells. Moreover, plasmablast cells showed a comparatively lower BCMA expression, a long term survival marker for plasma cells. Collectively, long term immunity is likely to be hampered, another observation being reported recently. The role of crucial immune cells identified in this study needs to be confirmed by functional assays.

In view of the involvement of multiple immune cell subsets and numerous markers, major findings of the study are outlined in Figure 7. In summary, while confirming previous findings, our study revealed that Indian patients exhibited a different set of immunological modulation in SARS CoV‐2 infection and identified additional prospective severity prognostic markers such as dendritic cells, activated CD8 T cells, IL‐2+ CD4 T cells, and follicular T helper cells. Confirmation in larger cohorts and in‐depth functional assays to understand the underlying mechanism(s) remains the way forward.

## CONFLICTS OF INTEREST

The authors declare no financial or commercial conflicts of interest.

## AUTHOR CONTRIBUTIONS

VAA and AK conceived and designed the study. SS was responsible for PRNT work. ACM reviewed the results and contributed to the manuscript. SP and SL were responsible for subject recruitment, collection of relevant clinical information and samples as well as follow up. All authors contributed to the article and approved the submitted version.

## ETHICAL STATEMENT

The entire study was approved by the Institutional Ethics Committee.

## Supporting information

Supporting information.

## Data Availability

The data that support the findings of this study are available from the corresponding author upon reasonable request.
