# Supplementary material for: Disease‐duration based comparison of subsets of immune cells in SARS CoV‐2 infected patients presenting with mild or severe symptoms identifies prognostic markers for severity
Source: Immun Inflamm Dis. 2021 Jan 16;9(2):419–34. doi: 10.1002/iid3.402 (PMC8014065; doi:10.1002/iid3.402)
Supplement: Supplementary file 1 — Supporting information. [file IID3-9-419-s001.docx]

|  | **Details of antibody panel (Clone & Dye )** | | | | |
| --- | --- | --- | --- | --- | --- |
|  |  | **Cellular markers/Proteins** | **Clone** | **Dye** | **Surface or Intracellular Marker** |
| **Ab Panel-1** | **CD4 & CD8 T cells** | CD3 | SK7 | APC | Surface |
|  |  | CD4 | SK3 | PE-Cy7 | Surface |
|  |  | CD8 | SK1 | FITC | Surface |
|  |  | HLA DR | LN3 | Per CP | Surface |
|  |  | CD38 | HIT2 | PE | Surface |
|  |  | CD40L | 24-31 | PE-Dazzle | Surface |
|  |  | IL-2 | MQ1 | BV785 | Intracellular |
| **Ab Panel-2** | **TFH cells** | CD3 | SK7 | APC | Surface |
|  |  | CD4 | SK3 | PE-Cy7 | Surface |
|  |  | CXCR5 | J25204 | FITC | Surface |
|  |  | PD-1 | EH12.2H7 | Per CP | Surface |
|  |  | ICOS | C398.4A | PE Dazzle | Surface |
|  |  | IL-21 | 3A3-N2 | PE | Intracellular |
| **Ab Panel-3** | **NK cells & monocytes** | CD3 | SK7 | Per CP | Surface |
|  |  | CD16 | 3g8 | FITC | Surface |
|  |  | CD56 | HCD56 | APC | Surface |
|  |  | CD107a | H4A3 | APC-Cy7 | Surface |
|  |  | IFN-γ | B27 | PE-Cy7 | Intracellular |
|  |  | CD14 | M5E2 | BUV395 | Surface |
| **AbPanel-4** | **B cells** | CD19 | HIB19 | APC | Surface |
|  |  | CD269 (BCMA) | 19F2 | PE-Dazzle | Surface |
|  |  | CD27 | M-T271 | FITC | Surface |
|  |  | CD360(IL-21R) | 2G1-K12 | PE | Surface |
|  |  | IgD/IgM | IA6-2/MHM-88 | Per CP | Surface |
|  |  | CD138 | MI15 | PE Cy7 | Surface |
| **Ab Panel-5** | **Dendritic cells** | Lineage Cocktail (CD3/14/16/19/20/56) |  | APC | Surface |
|  |  | HLA DR | LN3 | Per CP | Surface |
|  |  | CD1C | L161 | PE Cy7 | Surface |
|  |  | CD11C | APC -Cy7 | B415 | Surface |
|  |  | CD123 | 6H6 | PE | Surface |
|  |  | CD80 | L307.4 | BUV737 | Surface |
|  |  | CD86 | BU63 | FITC | Surface |
